# Oral microbiome changes in subjects with obesity following bariatric surgery compared to lean counterparts

**DOI:** 10.3389/fmicb.2025.1553404

**Published:** 2025-03-18

**Authors:** Keun-Suh Kim, Hee Sam Na, Tae Jung Oh, Hyejung Han, Jiyeon Kim, Jin-Sil Hong, Hyo-Jung Lee, Young Suk Park, Jin Chung

**Affiliations:** ^1^Department of Periodontology, Section of Dentistry, Seoul National University Bundang Hospital, Seongnam, Republic of Korea; ^2^Department of Oral Microbiology, School of Dentistry, Pusan National University, Yangsan, Republic of Korea; ^3^Department of Internal Medicine, Seoul National University Bundang Hospital, Seongnam, Republic of Korea; ^4^Department of Internal Medicine, Seoul National University College of Medicine, Seoul, Republic of Korea; ^5^Department of Surgery, Seoul National University Bundang Hospital, Seongnam, Republic of Korea

**Keywords:** obesity, bariatric surgery, oral microbiome, periodontal disease, saliva

## Abstract

**Introduction:**

This study aimed to compare oral microbiome profiles between obese and lean individuals without clinical periodontitis, and to assess changes in the oral microbiome of obese subjects following bariatric surgery.

**Methods:**

Individuals with a body mass index (BMI) > 30 were enrolled in the obese group, whereas those with a BMI < 23 served as controls. The obese surgery group, which consented to bariatric surgery, was followed up at 1, 3, and 6 months with clinical examinations. Oral examinations were conducted and periodontal disease was classified based on probing results. Saliva, buccal and subgingival microbiome samples were analyzed for community diversity, relative bacterial abundance, and differential abundance between control (*n* = 24) and obese group (*n* = 31). To evaluate effect size and statistical power, we used micropower, a simulation-based method for Permutational Multivariate Analysis of Variance-based *β*-diversity comparisons.

**Results:**

The obese group exhibited distinct alpha diversity (buccal: Chao1 p = 0.0002, Shannon *p* = 0.0003, supragingival: Shannon *p* < 0.0001) compared with the control group. Bray-Curtis distance analysis indicated significant disparities in microbiome composition distribution in saliva (*p* = 0.003), buccal (*p* = 0.002), and subgingival plaque samples (*p* = 0.001). Although the obese and normal weight groups exhibited no significant periodontal differences, the obese group showed distinct species associated with periodontal disease, especially in subgingival plaque including *Filifactor alocis*, *Peptostreptococcaceae* spp., *Prevotella* spp., and *Treponema maltophilum.* Cluster analysis of the obese surgery group indicated the emergence of microbiomes associated with a healthy state that increased over time including *Streptococcus salivarious* and various *Veillonella* spp., whereas clusters containing periodontal pathogens including *Porphyromonas* spp., tended to diminish.

**Discussion:**

The oral microbiome at 6 months post-bariatric surgery indicates a potential shift toward a healthy periodontal state, suggesting that weight loss interventions may positively impact oral microbial communities even in the absence of clinical periodontitis.

## 1 Introduction

In recent decades, the global prevalence of obesity has reached alarming levels and it has emerged as a significant public health concern. Obesity not only poses a substantial burden on an individual’s physical and psychological well-being but is also a pivotal risk factor for the development of various metabolic disorders, including type 2 diabetes mellitus, cardiovascular diseases, and certain cancers (Hruby and Hu, 2015; Arnold et al., 2015; Lauby-Secretan et al., 2016; GBD 2015 Obesity Collaborators et al., 2017).

Mounting evidence suggests that the gut microbiome plays a key role in the development and progression of obesity (Ridaura et al., 2013). The gut microbiota actively participates in energy regulation, nutrient metabolism, and the modulation of inflammation, thereby influencing host physiology and adiposity (Turnbaugh et al., 2006; Shen et al., 2013). The oral microbiome is the second largest microbiome community after the gut (NIH HMP Working Group et al., 2009) and has been well studied in relation to systemic diseases; however, it is comparatively less studied than the gut microbiome in relation to obesity. Recently, the oral microbiome has been shown to be associated with weight gain (Yang et al., 2019), and influence metabolic diseases, including obesity, through various mechanisms related to low-grade inflammation (Goodson et al., 2009; Gasmi Benahmed et al., 2021; Ganesan et al., 2021). Additionally, the oral microbiome, particularly species known as the “red complex,” has been strongly linked to periodontitis (Chen et al., 2018; Manzoor et al., 2024). Several studies have suggested a complex interplay between the oral microbiome, obesity, and periodontal health (reviewed by Reytor-González et al., 2024). Obesity has been associated with alterations in the oral microbiome composition, potentially predisposing individuals to periodontal disease. However, the exact mechanisms linking obesity, oral microbial dysbiosis, and periodontal health remain unclear, particularly in individuals without clinical periodontitis.

Bariatric surgery is an effective and durable method for weight loss in patients with severe obesity (Maciejewski et al., 2016; Schauer et al., 2017). Several studies have documented dynamic shifts in the gut microbiome (Furet et al., 2010; Liou et al., 2013) but few have investigated oral microbial diversity and abundance following bariatric surgery (Džunková et al., 2020; Stefura et al., 2022). There is insufficient research on whether surgical improvement of obesity in patients can lead to changes in the oral microbiome.

This study aimed to investigate significant differences in the oral microbiota between individuals of normal weight and those with obesity without clinically significant differences in periodontitis. Furthermore, we examined changes in the oral microbiota following surgical intervention.

## 2 Materials and methods

### 2.1 Study population and sample collection

**Figure 1:**
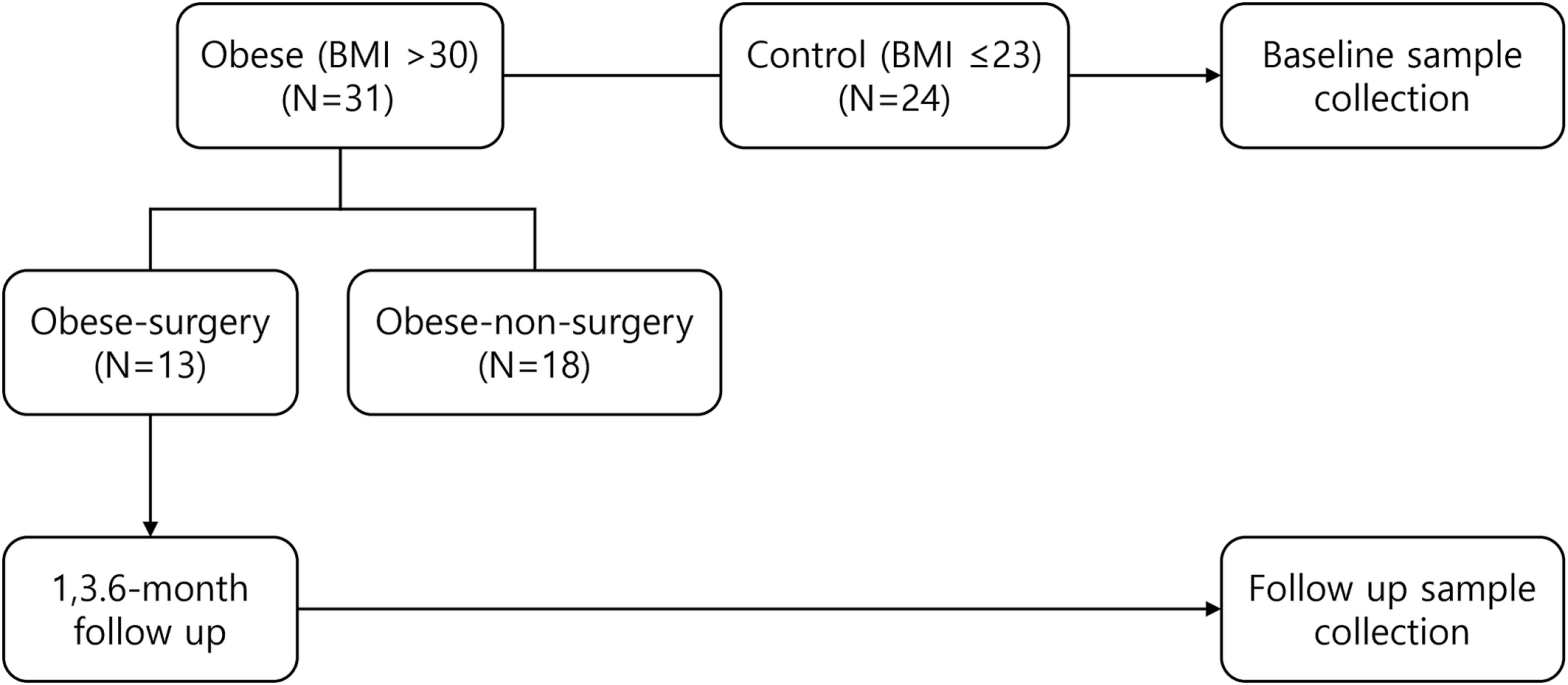
Flow of study population.

From September 2021 to March 2023, patients with a body mass index (BMI) > 30 were recruited as the test group (obese group), and those with a BMI < 23 were recruited as the control group. Patients in the obese group who consented to undergo bariatric surgery were referred to as the obese surgery (OS) group and were followed-up at 1, 3, and 6 months to collect clinical examination results, blood test results, and saliva and buccal swab samples (Figure 1). All participants provided written informed consent, and the study protocol was approved by the Institutional Review Board of Seoul National University Bundang Hospital (IRB No.: B-2001-586-303).

### 2.2 Oral sample collection

Subgingival plaque, saliva, and buccal swabs were collected from all the participants at the Department of Periodontology, Section of Dentistry, Seoul National University Bundang Hospital, Korea. Buccal swab samples were obtained from the mucosa of both cheeks, using a sterile swab kit (Kim et al., 2023). The cotton swab portion of the kit was immersed in a conical tube containing sterile distilled water for more than 20 s, and the tube was then sealed. Subgingival plaque samples were collected from the two deepest periodontal pocket sites of each participant’s teeth using a curette. The collected plaque was transferred onto the periopaper and sealed in a sterile conical tube for storage. Saliva was collected for 20 min without stimulation. Participants were requested to fast for 2 h and refrain from oral hygiene (brushing or flossing teeth) for 2 h before sampling. Samples from all subjects were collected and stored at −80°C for subsequent processing.

### 2.3 Oral examination

Oral examinations were performed by a professional periodontist. Participants were screened for other oral diseases such as dental caries and mucosal lesions. Those with active oral diseases other than periodontitis were excluded from the study. Panoramic radiographs were obtained and the number of missing teeth was counted. To classify periodontitis (PD), probing results were recorded at six sites for each tooth (Matuliene et al., 2008). Periodontal disease was classified according to the criteria of the American Academy of Periodontology/Centers for Disease Control and Prevention (Eke et al., 2012) and further categorized into the non-PD and PD groups based on the presence of at least one site with a probing pocket depth of ≥5 mm (McCracken et al., 2017). Patients were excluded if they had used steroids, immunosuppressants, rheumatic medications, or antibiotics in the past month.

### 2.4 Bariatric surgery procedures

All patients in the OS group underwent laparoscopic sleeve gastrectomy. Sleeve gastrectomy was performed by inserting four trocars into the abdominal cavity, followed by dissection of the greater curvature and fundus of the stomach using a laparoscopic bipolar device. Subsequently, the stomach was resected using a laparoscopic surgical stapler, starting 4 cm from the pylorus, and a 36Fr bougie was used for gastric calibration.

### 2.5 Extraction of genomic DNA and next-generation sequencing

Total DNA was extracted from the buccal and subgingival plaque using a Gram-positive DNA purification kit (Lucigen, Biosearch Technology, Novato, CA). For each saliva sample, 200 μL was centrifuged, and the resulting pellet was used for DNA extraction with a DNA extraction kit. Each sequenced sample was prepared according to the Illumina 16S Metagenomic Sequencing Library protocols to amplify the V1 and V2 region (27F-338R) (Na et al., 2023). DNA quality was measured using PicoGreen and NanoDrop ND-1000 spectrophotometer (Thermo Fisher Scientific, United States) and stored at −80°C until use. The purified amplicons were combined in equimolar amounts and subjected to paired-end sequencing using NovaSeq (Illumina, San Diego, CA, United States).

### 2.6 Bioinformatic analysis, statistical analysis, and visualization

Demographic characteristics were analyzed using *t*-tests, chi-square tests, and Fisher’s exact tests. The Wilcoxon rank-sum test was used to compare the clinical parameters of patients undergoing bariatric surgery. Continuous variables are expressed as medians with interquartile ranges, and categorical variables are presented as counts or percentages. Statistical significance was set at *p* < 0.05. Statistical analyses were performed using R ver. 4.1.2 (R Foundation for Statistical Computing, Vienna, Austria).

Basic microbiome analyses were performed using QIIME2 (version 2020.6) (Hall and Beiko, 2018) and the associated plugins. Choa1 and Shannon’s indices were used to measure alpha diversity. Principal coordinate analysis of the Bray–Curtis distance was performed to determine the community structure using the vegan package v2.3-0 in R software v3.2.1. The Kruskal–Wallis test and non-parametric permutation multivariate analysis of variance were used to assess the statistical significance of alpha and beta diversities, respectively. The species of each Operational Taxonomic Unit (OTU) was determined by a pre-trained Naive Bayes classifier, using the Human Oral Microbiome Database 16S rRNA Extended RefSeq sequences database (version 15.1) (Wade, 2013). Differential abundance of specific bacterial species in each group was assessed using the linear discriminant analysis effect size (LEfSe) (Segata et al., 2011), ANOVA-like Differential Expression (ALDEx2) (Fernandes et al., 2014), and Analysis of Compositions of Microbiomes (ANCOM) (Mandal et al., 2015) tools with their default settings. A linear discriminant analysis (LDA) score of 2.5 was used as the cut-off. Species identified as significant by at least two of these methods were considered reliable. To characterize the microbial variation pattern following bariatric treatment, the expression mode clustering was analyzed and visualized using the time course sequencing (“TCseq”) package (version 1.27.0).

## 3 Results

### 3.1 Participant characterization

We recruited 55 participants, of which 24 were in the control group (BMI <23) and 31 were in the obese group (BMI ≥30). The demographic characteristics of the participants are shown in Table 1. There were significant differences in BMI (*p* < 0.001), weight (*p* < 0.001), and smoking history (*p* = 0.017) between the two groups. Diabetes and hypertension were significantly more prevalent in the obese group than in the control group. There were no significant differences in the degree of periodontitis or tooth loss between the two groups. However, there was a significant difference in the mean probing pocket depth, which was within the normal range in both groups.

**Table 1:** Demographic characteristic of participants.

	Control (BMI < 23)	Obese (BMI ≥ 30)	*p*
	(*N* = 24)	(*N* = 31)	
Age (years)	37.0 [30.0, 45.5]	35.0 [29.5, 39.5]	0.444
Sex			
Male	24 (100.0%)	31 (100.0%)	
BMI^a^ (kg/m^2^)	21.8 [20.3, 22.5]	34.9 [31.8, 40.6]	<0.001**
Weight (kg)	65.7 [62.2, 69.9]	108.8 [95.6, 123.2]	<0.001**
Smoking			0.017*
Current	2 (8.3%)	11 (35.5%)	
Never	19 (79.2%)	13 (41.9%)	
Ever	3 (12.5%)	7 (22.6%)	
Diabetes			0.012*
Yes	0 (0.0%)	9 (29.0%)	
No	24 (100.0%)	22 (71.0%)	
Hyperlipidemia			0.294
Yes	2 (8.3%)	7 (22.6%)	
No	22 (91.7%)	24 (77.4%)	
Hypertension			<0.001**
Yes	0 (0.0%)	18 (58.1%)	
No	24 (100.0%)	13 (41.9%)	
Periodontitis (AAP/CDC^b^)			0.109
No	8 (33.3%)	4 (12.9%)	
Mild	0 (0.0%)	3 (9.7%)	
Moderate	13 (54.2%)	22 (71.0%)	
Severe	3 (12.5%)	2 (6.5%)	
Periodontitis≥5 mm^c^			0.442
Yes	9 (37.5%)	16 (51.6%)	
No	15 (62.5%)	15 (48.4%)	
Tooth loss (*N*)	0.0 [0.0, 1.5]	0.0 [0.0, 0.0]	0.102
Mean PPD^d^ (mm)	2.3 [2.1, 2.5]	2.5 [2.3, 2.7]	0.035*

a BMI, body mass index.

b AAP/CDC, classification of American Academy of Periodontology/Centers for Disease Control and Prevention.

c Periodontitis_ > 5 mm, presence of at least one site with a probing pocket depth of 5 mm or more.

d PPD, probing pocket depth.

* *p* < 0.05; ** *p* < 0.001.

### 3.2 Diversity and abundance of microbiota in the control and obese groups

**Figure 2:**
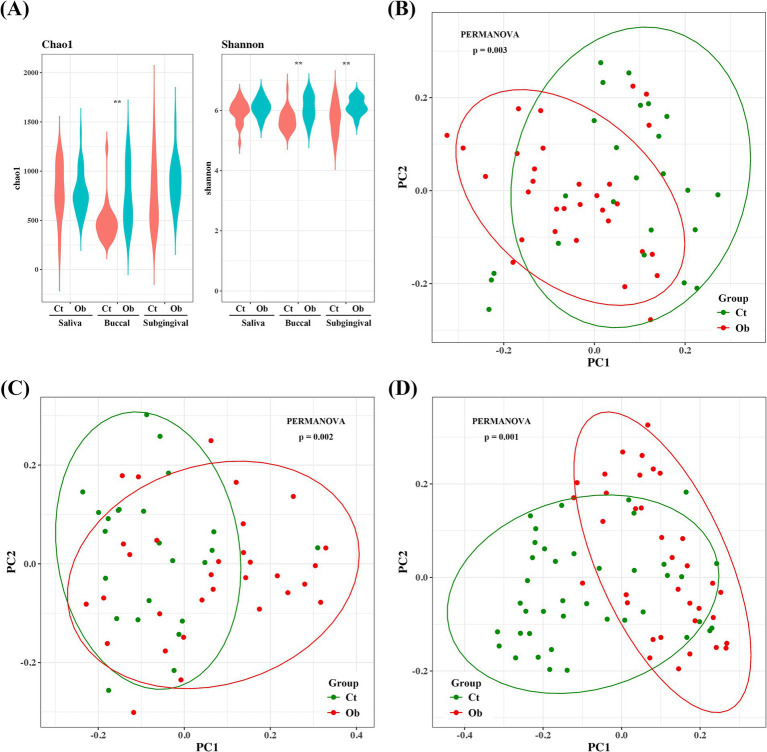
Bacterial community comparison between the obese and control groups. **(A)** Alpha diversities (Chao1 and Shannon indices) of subgingival plaque, saliva, and buccal samples. Beta diversities of **(B)** saliva, **(C)** buccal swab, and **(D)** plaque samples. ** *p* < 0.01.

The alpha diversity of the microbiota was estimated using the Chao1 and Shannon indices. Buccal swab samples from the obese group had significantly higher Chao1 and Shannon indices. The Shannon index of the plaque samples was also significantly higher in the obese group (Figure 2A). For principal coordinate analysis, the Bray–Curtis distance was used to analyze the distribution of the microbiota. The microbiome compositions of saliva, buccal swabs, and subgingival samples were significantly different between the control and obese groups (Figures 2B–D).

**Figure 3:**
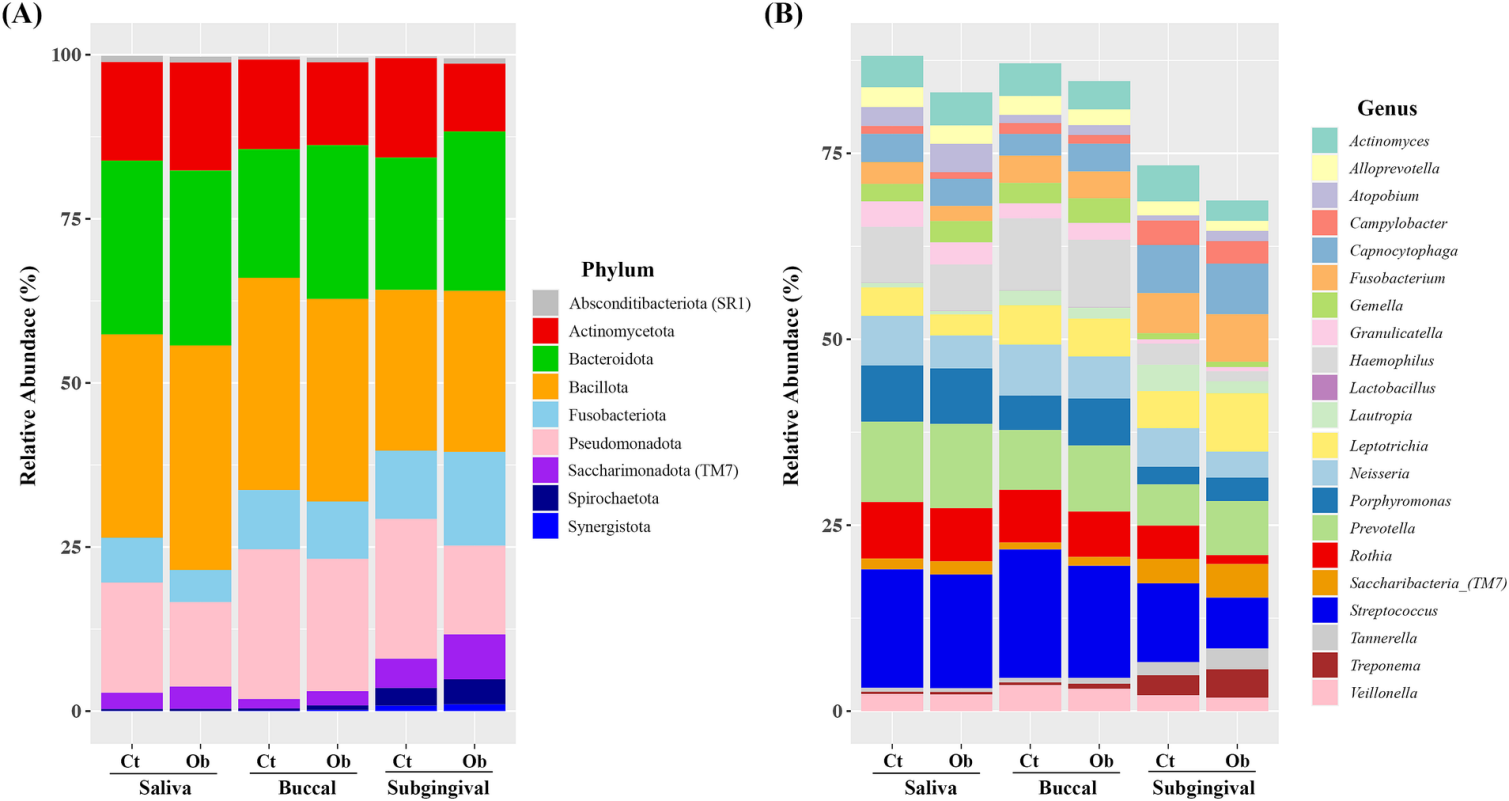
Relative abundance of bacterial species in saliva, buccal, and subgingival plaque samples of the obese and control groups. **(A)** Phylum level, **(B)** genus level. Cr, control group; Ob, obese group.

When the relative abundances were analyzed, the five most abundant phyla were Bacillota, Bacteroidota, Actinomycetota, Pseudomonadota, and Fusobacteriota, representing more than 95% of the total taxa in the saliva and buccal swabs. In subgingival plaque samples, these five phyla accounted for most taxa in both groups. Interestingly, in the subgingival plaque samples, there was an increase in the abundance of Fusobacteriota (control vs. obese: 10.39% vs. 14.27%), Candidatus Saccharimonadota (4.45% vs. 6.83%), and Spirochaetota (0.29% vs. 0.80%), with a higher prevalence observed in the obese group (Figure 3A).

The six most abundant genera in the saliva and buccal swab samples were *Streptococcus*, *Prevotella, Hemophilus*, *Rothia*, *Porphyromonas,* and *Neisseria* accounting for more than 51% of the total taxa in both groups (Figure 3B). In the subgingival plaque samples, *Streptococcus, Capnocytophaga, Leptotrichia, Prevotella, Fusobacterium, Neisseria,* and *Saccharibacteria (TM7)* were the most abundant genera, representing more than 43% of the total taxa.

The average abundance of *Neisseria* (6.66% vs. 4.43%) was higher in saliva samples from the control group. In the buccal swab samples, the abundance of *Porphyromonas* (4.63% vs. 6.32%), *Capnocytophaga* (2.91% vs. 3.73%), and *Saccharibacteria (TM7)* (0.93% vs. 1.19%) was higher in the obese group. In subgingival plaque samples, the abundance of *Prevotella* (5.54% vs. 7.27%), *Fusobacterium* (5.40% vs. 6.39%), *Tannerella* (1.74% vs. 2.79%), *Lepiotrichia* (4.99% vs. 7.86%), *Saccharibacteria (TM7)* (3.27% vs. 4.51%), *Porphyromonas* (2.37% vs. 3.16%), and *Treponema* (2.69% vs. 3.82%) was higher in the obese group. The distribution of major phyla and genera in the control and obese groups, separately for saliva, buccal swabs, and subgingival samples, is presented in the Supplementary Figures 1, 2.

### 3.3 Species taxa comparison between the control and obese groups

**Figure 4:**
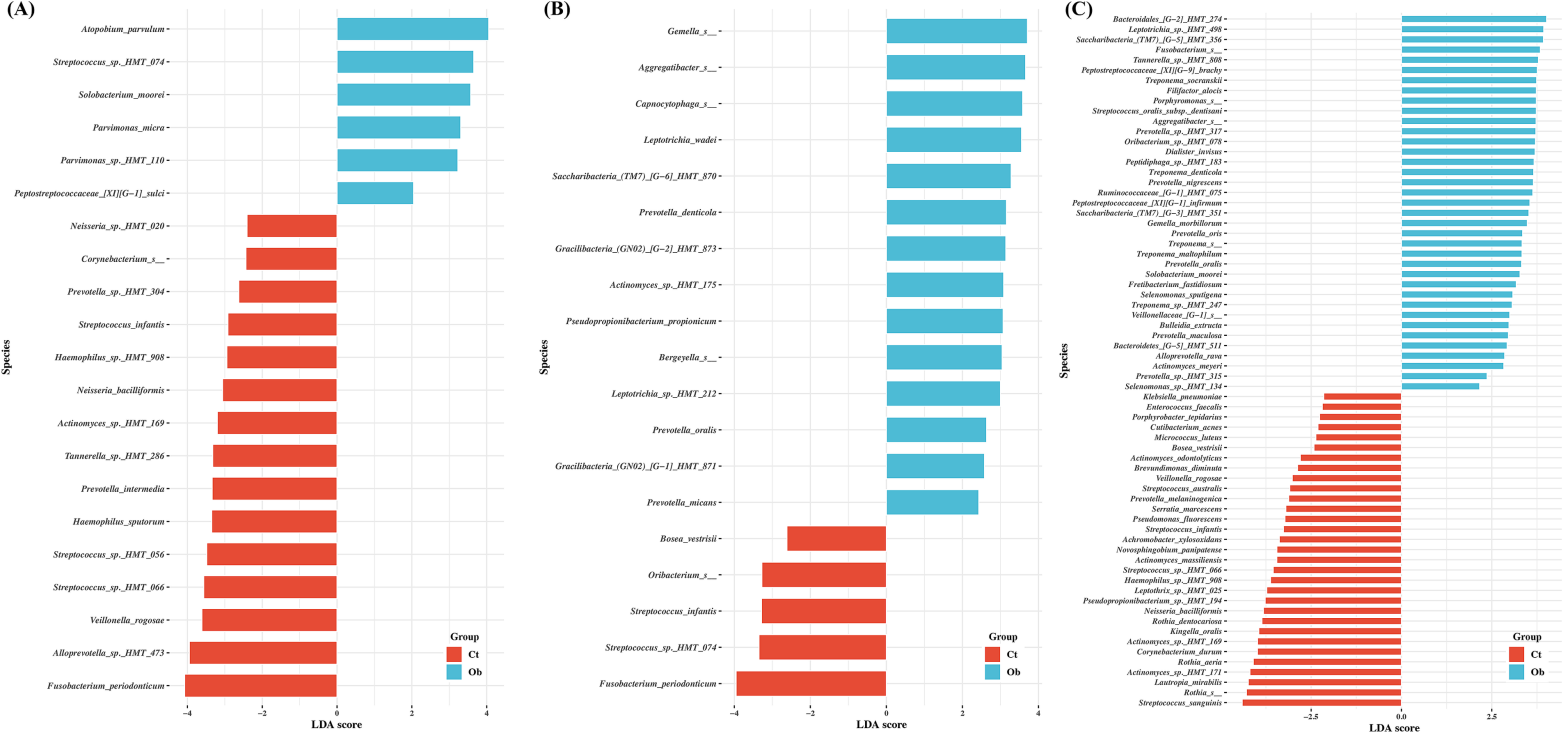
Comparisons of the taxa in control and obese group samples showing significant differences depending on sampling sites. **(A)** saliva, **(B)** buccal, **(C)** subgingival plaque. Linear discriminant analysis (LDA) and effect size analysis (LEfSe) was performed for the analysis. LDA score > 2 was plotted.

LEfSe, ALDEx2, and ANCOM were used to evaluate the differences in bacterial species abundance between the obese and control groups. The number of significant species determined by LEfSe in the saliva, buccal swab, and subgingival plaque samples was 38, 65, and 113, respectively (Figures 4A–C). In the control group, *Actinomyces* sp. *HMT-169, Fusobacterium periodonticum, Haemophilus* sp. *HMT-908, Neisseria bacilliformis, Streptococcus infantis, Streptococcus* sp. *HMT-066,* and *Veillonella rogosae* were significant in at least two sampling sites. In the obese group, *Prevotella micans, Prevotella oralis* and *Solobacterium moorei* were significant in at least two sampling sites. Additionally, analysis of subgingival plaque samples identified several species that were significantly different between the control and obese groups, including *Aggregatibacter* spp.*, Leptotrichia* spp.*, Peptostreptococcaceae* spp.*, Prevotella* spp.*, and Treponema* spp. (Figure 4C). Interestingly, *T. socranskii, T. denticola,* and *Filifactor alocis,* well-known periodontopathogens, were among the most significant species found in the subgingival plaque samples of the obese group.

### 3.4 Change in clinical characteristics after bariatric surgery

Overall, 13 patients in the obese group underwent bariatric surgery. Upon clinical evaluations of these 13 individuals, remarkable and statistically significant decreases were observed in the BMI and hemoglobin A1c, aspartate aminotransferase, and alanine aminotransferase levels between baseline and 6-month values. While total cholesterol levels did not show a significant difference, triglyceride and high-density lipoprotein cholesterol levels significantly decreased and increased, respectively (Table 2).

**Table 2:** Clinical characteristics after bariatric surgery at baseline and 1, 3, and 6-month follow-up.

	Baseline	1 month	3 months	6 months	*p* ^a^
	(*N* = 13)	(*N* = 13)	(*N* = 12)	(*N* = 10)	
BMI	41.1 ± 7.0	35.5 ± 6.0	32.1 ± 5.3	29.3 ± 4.8	<0.001^**^
Weight	130.7 ± 26.7	111.0 ± 22.8	99.0 ± 19.9	90.0 ± 17.6	<0.001^**^
Glucose	107.4 ± 13.6	90.2 ± 7.9	–	93.2 ± 6.8	0.004^*^
HbA1c	6.1 ± 1.2	5.4 ± 0.4	5.3 ± 0.2	5.1 ± 0.3	0.002^*^
Cholesterol	197.6 ± 39.7	166.8 ± 46.1	201.8 ± 37.2	199.9 ± 19.9	0.859
TG	197.0 ± 123.5	92.4 ± 23.8	105.8 ± 11.8	93.4 ± 17.9	0.011^*^
LDL	123.9 ± 30.4	113.0 ± 52.1	157.0 ± 39.1	127.2 ± 18.1	0.775
HDL	45.6 ± 9.3	41.2 ± 9.8	105.0 ± 133.8	54.9 ± 6.8	0.019^*^
AST	62.1 ± 37.0	36.8 ± 20.2	26.1 ± 11.5	23.9 ± 9.3	<0.001^**^
ALT	119.3 ± 68.0	58.8 ± 42.3	30.4 ± 25.0	19.5 ± 7.1	<0.001^**^

BMI, body mass index; HbA1c, hemoglobin A1c; TG, triglyceride; LDL, low density lipoprotein; HDL, high density lipoprotein; AST, aspartate aminotransferase; ALT, alanine aminotransferase.

a *p-value is calculated by comparing baseline and 6-month results.*

* *p* < 0.05; ** *p* < 0.001.

### 3.5 Changes in the microbiome after surgery in the OS group

**Figure 5:**
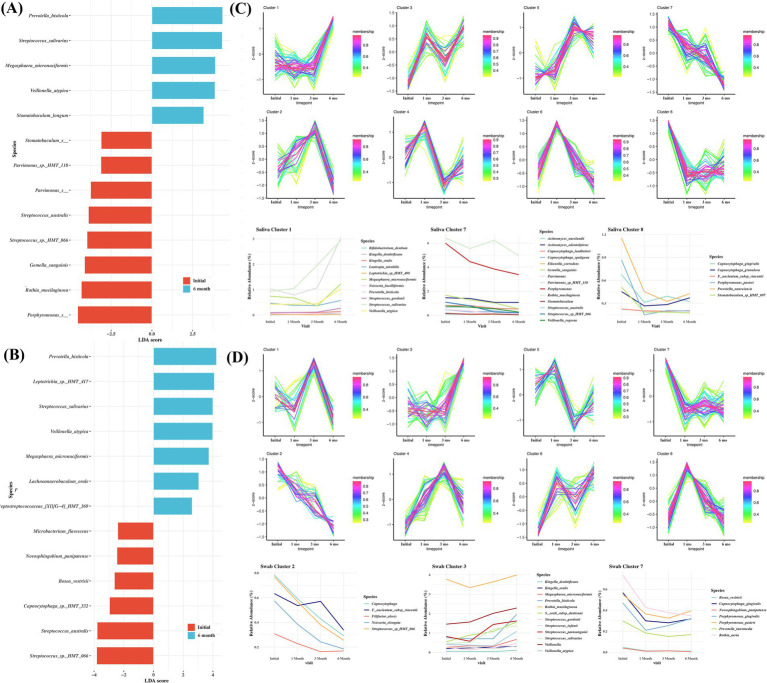
Comparisons of the taxa in samples obtained at baseline and 6-month follow-up from the surgical treatment group, showing significant differences depending on sampling sites. **(A)** saliva, **(B)** buccal. Linear discriminant analysis (LDA) and effect size analysis (LEfSe) was performed for the analysis. LDA score > 2 was plotted. Time course sequencing (TCseq) analysis in saliva **(C)** and swab **(D)** following surgical treatment.

To evaluate the changes in the microbiome following surgical treatment, saliva and buccal samples were collected for up to 6 months. To simplify the comparison, microbiomes were compared between samples obtained at the initial visit and after 6 months. In saliva samples, *Porphyromonas* spp.*, Rothia mucilaginosa, Gemella sanguinis,* and *Streptococcus* sp. *HMT-066* were among the most significant species at the initial visit. At 6-month follow-up, *Provotella histicolla, Streptococcus salivarious, Megasphaera micronuciformis,* and *Veillonella atypica* were among the most significant taxa found in the saliva (Figure 5A). In buccal swab samples, *Streptococcus sp_HMT_066, Streptococcus australis,* and *Capnocytophaga* sp. *HMT-332* were among the most significant species at the initial visit. At 6-month follow-up, *Prevotella histicola, Leptotrichia* sp. *HMT-417, Streptococcus salivarius,* and *Veillonalla atypica* were the among the significant species (Figure 5B).

To further characterize the variation pattern of microbes following surgical treatment, TCseq analysis was performed, and eight clusters were determined as the ideal grouping strategy. Microbes from saliva cluster 1 exhibited an increasing trend. These include *Bifidobacterium dentium, K. oralis, K. dentrificans, L. mirabilis, N. bacilliformis,* and *Streptococcus* spp. The microbes in saliva clusters 7 and 8 exhibited a decreasing trend. These included *Actinomyces naeslundii, A. odontolyticus, Capnocytophaga leadbetteri, C. gingivalis, C. granulosum, C. sputigena, Eikenella corrodens,* and *Porphyromonas pasteri* (Figure 5C). In cluster 1 from buccal swab samples, *K. oralis, K. dentrificans,* and *various Streptococcus* spp. showed an increasing pattern. In clusters 2 and 7 from buccal swab samples, the abundance of *F. alocis, Capnocytophaga, Fusobacterium nucleautum subsp. Vincentii,* and *P. gingivalis* showed a decreasing pattern (Figure 5D).

## 4 Discussion

In this study, we compared the oral microbiomes in the saliva, buccal swabs, and subgingival plaque of obese and control groups and analyzed changes in the oral microbiome following bariatric surgery. The obese group was more enriched with microbes known to be periodontopathogens, including *Filifactor alocis, Treponema* spp.*, T. socranskii*, and *T. denticola*, at least at two sampling sites, than the control group. Additionally, cluster analysis of the OS group identified clusters of healthy state–related microbiomes that increased over time, and clusters of periopathogen-related microbiomes that tended to decrease.

Studies on the association between the oral microbiome and obesity have shown that the composition of the oral microbiome differs between healthy individuals and those with obesity at different sampling sites (Mathur and Barlow, 2015; Koliada et al., 2017; Tam et al., 2018). Compared with saliva or buccal swab samples, we found that there were a greater number of genera in the subgingival plaque samples that showed differences between the obese and control groups (Figure 3), suggesting that the subgingival plaque microbiome may be more useful in explaining obesity than that of other sites. Additionally, compared to the control group, the obese group showed a higher proportion of *Porphyromonas, Capnocytophaga*, and *Saccharibacteria (TM7)* in buccal swab samples, whereas *Prevotella, Fusobacterium, Tannerella, Lepiotrichia, Saccharibacteria (TM7), Porphyromonas*, and *Treponema* were more abundant in subgingival plaque samples. *Porphyromonas, Fusobacterium*, *Capnocytophaga*, *Prevotella, Tannerella*, and *Treponema* are genera commonly associated with periodontopathogens (Socransky et al., 1998; Darby and Curtis, 2001; Mineoka et al., 2008).

The number of species varied significantly, with the highest number found in subgingival plaques, followed by buccal swabs and saliva (Figure 4), suggesting that the environment in the subgingival space differed between the obese and control groups. This implies that the subgingival space may be more affected by systemic conditions than other sampling sites, which may lead to more differences. In the saliva of the obese group, *Prevotella micans, Prevotella oralis* and *Solobacterium moorei* were significant in at least two samples. In addition, *T. socranskii, T. denticola,* and *Filifactor alocis* were among the most significant species found in the subgingival plaque samples of the obese group, *Prevotella oralis, and Prevotella* sp. *HMT-315* (Nadkarni et al., 2012) are conventional periodontal pathogens. *S. moorei* and *F. alocis* are involved in the onset and progression of periodontitis (Hiranmayi et al., 2017). The abundance of *Saccharibacteria (TM7) HMT-351* is associated with periodontitis; however, the mechanism is not clear (Baker et al., 2024). In contrast, in the control group, *Actinomyces* sp. *HMT-169, Fusobacterium periodonticum*, and *Haemophilus* sp. *HMT-908* were significant, which are commonly associated with periodontal health or gingivitis (Zaura et al., 2009; Abusleme et al., 2021).

Despite the absence of significant differences in clinical periodontitis, the obese and control groups exhibited differences in oral microbiome composition, especially in the subgingival plaque samples. These findings suggest a strong association between obesity and periodontitis. Three of the eight longitudinal studies included in this systematic review reported a direct association between obesity and the development of periodontitis (Keller et al., 2015). Our study provides evidence of an oral microbiome base that support the associations found in these studies. The species that were significantly increased in the obese group (Figure 4) were present in relatively low proportions in the overall microbiome; however, they were associated with the onset of periodontal disease. Therefore, although periodontal disease is currently mild in these individuals, its severity is likely to increase in the future. This is also related to the finding that periodontal disease and the healthy state do not differ significantly in the type of microbiome. The difference between the healthy state, gingivitis, and periodontitis lies in the frequency or composition ratio of the microbiome (Chen et al., 2018; Abusleme et al., 2021).

The possibility of a close relationship between obesity and the oral microbiome was explored by comparing the pre-and post-bariatric surgery microbiomes, which revealed that post-bariatric surgery saliva and buccal samples were dominated by healthy and gingivitis-associated microbiomes (Figures 5A,B). There was a gradual change in the microbiome at 3- and 6-months post-surgery, which was more clearly observed through cluster analysis. Cluster analysis of the microbiome, categorized according to decreasing or increasing patterns, was performed using saliva and buccal swabs (Figures 5C,D). In the swab samples, clusters that decreased after surgery included species associated with periodontitis such as *Filifactor alocis, Fusobacterium nucleatum subsp. vincentii, Porphyromonas gingivalis*, and *Prevotella intermedia*. Clusters that increased after surgery included *Kingella, Streptococcus, Rothia,* and *Veillonella* spp., which are commensal microbes found in a healthy state. *Veillonella* spp. can serve as bridges or commensal species in the absence of periodontitis (Zhou et al., 2021).

In saliva samples, a decrease after surgery was observed in clusters 7 and 8, whereas an increase was observed in cluster 1. Among the species that show a tendency to decrease, *Porphyromonas, Stomatobaculum* spp., and *Porphyromonas pasteri* are species associated with periodontitis, while *Prevotella histicola*, which shows a significant increase in saliva cluster 1, is associated with healthy subjects (Moritani et al., 2015). Although the trending species in the saliva and buccal swab samples were not the same, we observed a decrease in the microbiome species associated with periodontitis after bariatric surgery, along with an improvement in obesity-related clinical examination values, suggesting that there may be a link between obesity and periodontal disease through the oral microbiome.

There are notable differences between the outcomes of bariatric surgery and medications such as metformin and statins in terms of oral microbiome alteration and clinical enhancement. Metformin, commonly used for diabetes management, has been shown to decrease the relative proportion of disease-related oral microbiota (Sun et al., 2020). Similarly, statins used for hyperlipidemia are associated with minor changes in oral microbial composition (Kamińska et al., 2019; DeClercq et al., 2021). However, these medications exhibit limited effects on reshaping the oral microbiome toward a healthier state. With the emergence of novel weight loss medications, such as GLP-1 receptor agonists, the paradigm of obesity treatment is rapidly evolving. These pharmacological agents provide non-surgical options for significant weight loss, potentially challenging the traditional dominance of bariatric surgery. This shift underscores the importance of investigating how different methods of weight loss—surgical or pharmacological—affect the oral microbiota. Understanding how quantitative changes in body weight led to qualitative shifts in microbial communities presents an intriguing avenue for future research. While our findings suggest that bariatric surgery induces more substantial shifts in the oral microbiome compared to medication-based approaches, further studies are needed to confirm these effects. The observed alterations in microbial composition post-surgery, coupled with clinical improvements, highlight the potential of surgical interventions to influence both metabolic and oral health outcomes.

Our study had several limitations. First, the number of subjects in the bariatric surgery group was not large enough, and additional subject loss during the follow-up period limited our ability to identify more than a trend toward changes in the microbiome after surgery. While the Control and Obese groups had an adequate number of subjects for microbiome analysis, the bariatric surgery group had a relatively small sample size, which tended to be insufficient for achieving reliable statistical power (Kelly et al., 2015). In general, the identification of species from an insufficient number of analyzed samples may have limitations, as many oral microbiomes can be present in both symbiotic and dysbiotic situations in a healthy state, gingivitis, or periodontitis (Colombo and Tanner, 2019). However, our observations suggest that as obesity improves, there appears to be a trend toward changes in the oral microbiome that may potentially reduce the abundance of species associated with dysbiosis and periodontal disease. Second, we were unable to obtain subgingival plaque samples after surgery because the OS group did not visit a dentist; this requires further investigation. Third, another limitation of this study is the lack of dietary data and caries data for the obese patient group. Pre-surgery high intake of carbohydrates and fats, as well as post-surgery dietary changes, can influence not only obesity but also the composition of the oral microbiome (Millen et al., 2022). Recent research by Millen *et al.* has shown that fermentable carbohydrates (e.g., sucrose) promote the growth of cariogenic oral bacteria such as *Streptococcus mutans*. However, with the exception of *Leptotrichia* spp., this study did not find significant effects on the periodontal disease-associated microbiomes that showed changes in our research. Future research should be designed to incorporate dietary changes and analyze their association with microbiome alterations. This would provide a more comprehensive understanding of the relationship between diet, obesity, and oral microbiome composition. In addition, the smoking rate in the obese group showed a significant difference, which could potentially influence the study results. In our study, *Capnocytophaga* in buccal swab samples and *Leptotrichia* in subgingival plaque samples were genera that were more prevalent in the obese group. These genera have also been reported in other studies as being dominant in smokers (Wu et al., 2016). While it cannot be conclusively stated that smoking had the greatest impact on the oral microbiome characteristics of the obese group, it is clear that smoking is a factor that can influence the oral microbiome. Future studies should consider this when designing their research.

Notwithstanding, this is the first study to identify changes in periodontitis-associated bacteria in saliva and buccal swabs after bariatric surgery, and notably, these bacteria showed a significant decrease over time.

In summary, a comparison of the oral microbiota of obese and normal-weight groups without significant differences in clinical periodontal disease showed differences in the oral microbiome especially in the subgingival plaque samples, with a notable presence of species related to periodontal disease. Oral microbiome cluster analysis 6 months after bariatric surgery indicated the potential for changes in the oral microbiome toward a healthy state. Consequently, individuals with obesity need careful oral health management through regular check-ups to prevent periodontitis.

## Data Availability

The datasets presented in this study can be found in online repositories. The names of the repository/repositories and accession number(s) can be found at: https://www.ncbi.nlm.nih.gov/, PRJEB74337.

## Supplementary material

SUPPLEMENTARY FIGURE 1 Relative abundance of major phyla.

SUPPLEMENTARY FIGURE 2 Relative abundance of major genera.
