# Supplementary figures and images for: Oral microbiome changes in subjects with obesity following bariatric surgery compared to lean counterparts
Source: Front Microbiol. 2025 Mar 18;16:1553404. doi: 10.3389/fmicb.2025.1553404 (PMC11959278; doi:10.3389/fmicb.2025.1553404)

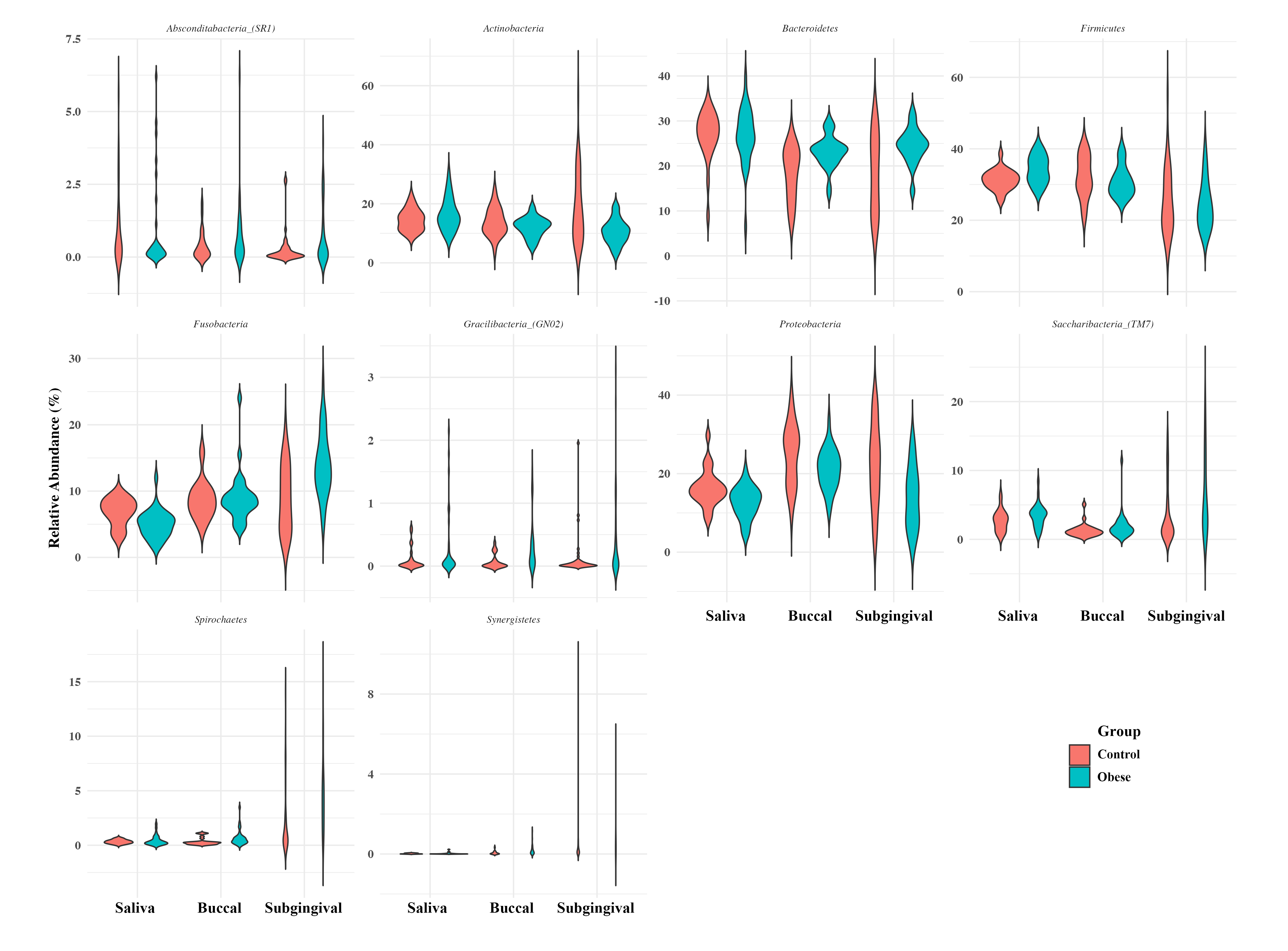

Supplement: SUPPLEMENTARY FIGURE 1 — Relative abundance of major phyla. [file Image_1.tif]

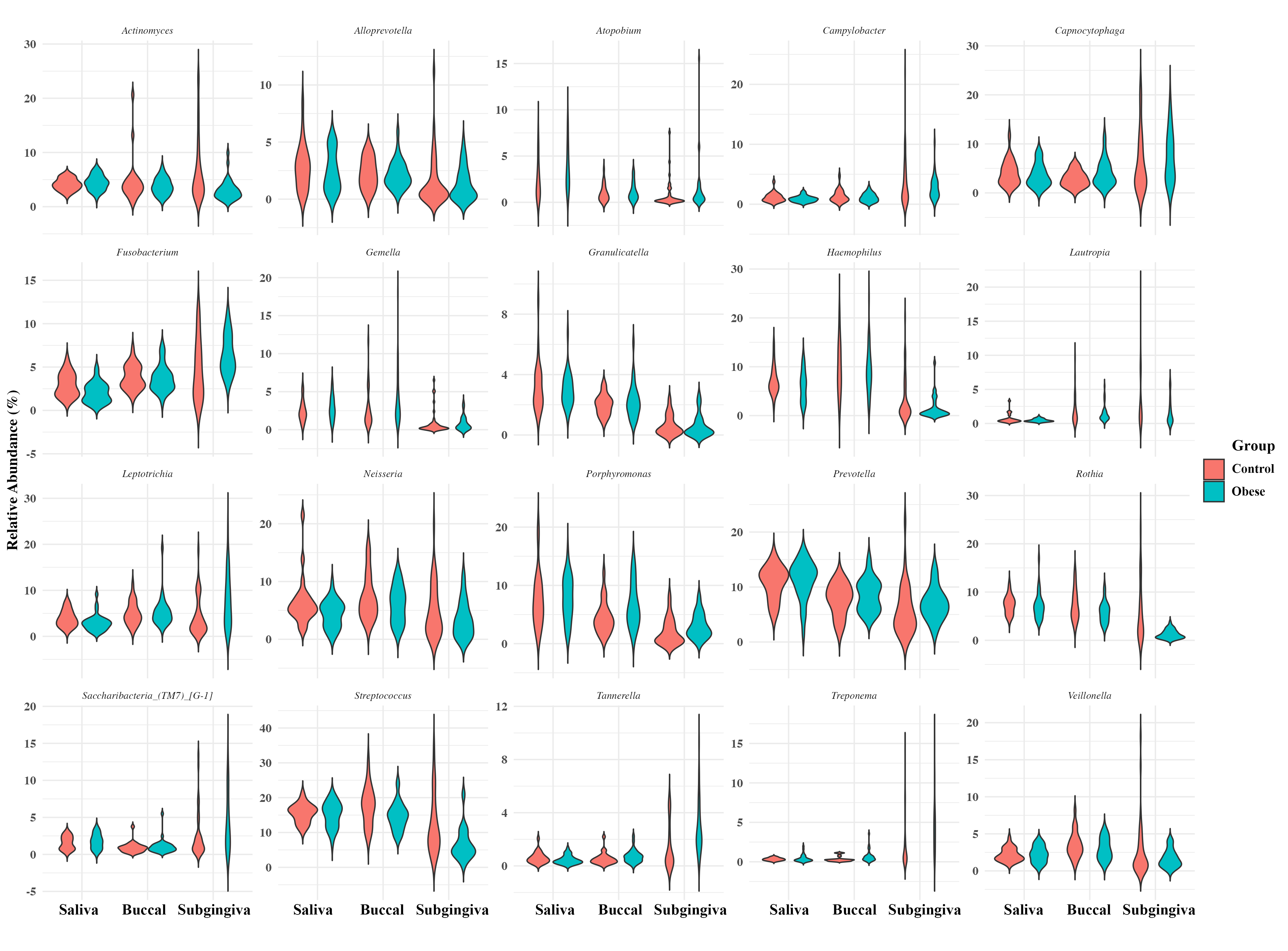

Supplement: SUPPLEMENTARY FIGURE 2 — Relative abundance of major genera. [file Image_2.tif]
